# Formulation Development and Evaluation of Fast Disintegrating Tablet of Cetirizine Hydrochloride: A Novel Drug Delivery for Pediatrics and Geriatrics

**DOI:** 10.1155/2014/808167

**Published:** 2014-02-18

**Authors:** Deepak Sharma, Mankaran Singh, Dinesh Kumar, Gurmeet Singh

**Affiliations:** ^1^Department of Pharmaceutics, Rayat Bahra Institute of Pharmacy, Hoshiarpur-146001, Punjab, India; ^2^Quantum Solutions, Chandigarh, Punjab, India; ^3^CSIR Institute of Microbial Technology, Chandigarh, Punjab, India; ^4^CT Institute of Pharmaceutical Sciences, Jalandhar-144020, Punjab, India

## Abstract

Recent developments in fast disintegrating tablets have brought convenience in dosing to pediatric and elderly patients who have trouble in swallowing tablets. The objective of the present study was to prepare the fast disintegrating tablet of Cetirizine Hydrochloride for allergic and respiratory disorders. As precision of dosing and patient's compliance become important prerequisite for a long-term treatment, there is a need to develop a formulation for this drug which overcomes problems such as difficulty in swallowing, inconvenience in administration while travelling, and patient's acceptability. Hence, the present investigation was undertaken with a view to develop a fast disintegrating tablet of Cetirizine Hydrochloride which offers a new range of products having desired characteristics and intended benefits. Superdisintegrants such as Sodium Starch Glycolate were optimized. Different binders were optimized along with optimized superdisintegrant concentration. The tablets were prepared by direct compression technique. The tablets were evaluated for hardness, friability, weight variation, wetting time, disintegration time and uniformity of content. Optimized formulation was evaluated by in vitro dissolution test, drug excipient compatibility and accelerated stability study. It was concluded that fast disintegrating tablets of Cetirizine Hydrochloride were formulated successfully with desired characteristics which disintegrated rapidly, provide rapid onset of action, and enhance the patient convenience and compliance.

## 1. Introduction

In spite of the increased focus and interest generated in the area of controlled release and targeted drug delivery system in recent years, tablet dosage forms that are intended to be swallowed whole, disintegrate, and release their medicaments rapidly in the gastrointestinal tract still remain the formulation of choice from both a manufacturing as well as a patient acceptability point of view. Thus, a drug given in the form of a tablet must undergo dissolution before being absorbed and eventually transported into systemic circulation [1]. Difficulties with and resistance to tablet-taking are most common in all patient groups and can exacerbate compliance problems and undermine treatment efficacy. Physical problems with swallowing (dysphasia) can occur at any age but are particularly prevalent in geriatric, pediatric, and psychiatric patients. Nonetheless, oral dosing remains the preferred mode of administration for many types of medication due to its simplicity, versatility, convenience, and patient acceptability [2]. By considering the above points, patient convenience and compliance oriented research has resulted in bringing out safer and newer drug delivery systems; one of such approaches is fast disintegrating drug delivery system [3]. Fast disintegrating drug delivery systems (FDDDS) are a new generation of formulations which combine the advantages of both liquid and conventional tablet formulations and, at the same time, offer added advantages over both traditional dosage forms. They provide the convenience of a tablet formulation and also allow the ease of swallowing provided by a liquid formulation. FDDDS offer the luxury of much more accurate dosing than the primary alternative, oral liquids [4]. Recent advances in novel drug delivery systems (NDDS) aim at enhancing the safety of a drug molecule while maintaining its therapeutic efficacy so as to achieve better patient compliance [5]. US Food and Drug Administration Center for Drug Evaluation and Research (CDER) defines, in the “Orange Book,” a FDT as “*a solid dosage form containing medicinal substances, which disintegrates rapidly, usually within a matter of seconds, when placed upon the tongue*”. European Pharmacopoeia described FDTs as “*uncoated tablets intended to be placed in the mouth where they disperse rapidly before being swallowed”* and as *tablets which should disintegrate within 3 minutes* [6]. Fast disintegrating tablets (FDT) are also known as fast dissolving, mouth dissolving, rapid-dissolving, quick disintegrating, orally disintegrating, rapimelt, fast melt, orodispersible, melt-in-mouth, quick dissolving, porous tablets, and EFVDAS (Effervescent Drug Absorption System) [7]. The bioavailability of drugs may be increased due to absorption of drug in oral cavity and also due to pregastric absorption of saliva containing dispersed drugs that pass down into the stomach. Moreover, the amount of drug that is subjected to the first pass metabolism is reduced as compared to standard tablet [8]. Formulation of the drug chosen for the treatment of allergic cough and other respiratory disorders is available in market in conventional tablet and liquid dosage forms. Liquid dosage forms are having their own limitation from stability and dose measurement perspectives. Tablets to be swallowed are resisted by pediatric patients and patient compliance is an issue with such dosage forms. Hence they do not comply with the prescription, which results in high incidence of noncompliance and ineffective therapy. The benefits, in terms of patient compliance, rapid onset of action, increased bioavailability, and good stability make fast disintegrating tablets popular as a dosage form of choice in the current market [9]. Cetirizine Hydrochloride is the active metabolite of the piperazine H_1_-receptor antagonist Hydroxyzine. It is a nonsedative second generation antihistamine drug used in the treatment of seasonal allergic rhinitis, perennial allergic rhinitis, chronic urticaria, and atopic dermatitis and also used as adjuvant in seasonal asthma and allergic cough. Cetirizine inhibits the release of histamine and of cytotoxic mediators from platelets, as well as eosinophil chemotaxis during the secondary phase of allergic response. Due to sore throat conditions, the patient experiences difficulty in swallowing a tablet type of dosage form. Thus, fast disintegrating tablets would serve as an ideal dosage form pediatric patients who find it difficult to swallow the conventional tablets and capsules [10]. Hence an attempt was made for preparation of fast disintegrating tablet of Cetirizine Hydrochloride with an aim of improving/enhancing patient convenience and compliance, reducing the lag time and providing faster onset of action to relieve the allergic and respiratory disorders immediately. 

## 2. Materials and Methods

### 2.1. Materials

Cetirizine Hydrochloride was received as gift sample from Trojan Pharma, Baddi, India. Microcrystalline Cellulose (Avicel PH-102) was obtained as gift sample from NB Entrepreneurs, Nagpur, India. Sodium Starch Glycolate (Primogel, Explotab) and directly compressible Mannitol (D-Mannitol) were purchased from Qualikems Fine Chem Pvt. Ltd. Sodium Srearyl Fumarate was Purchased from Himedia. Sodium Saccharin was purchased from Loba Chemie, Mumbai, and Talc from Nice Chemicals Private Limited, Hyderabad, India. All other chemicals and reagents which were of analytical grade were used.

### 2.2. Methods

#### 2.2.1. Selection of Excipients and Optimization of Their Concentration

The most important parameter that needs to be optimized in the development of fast disintegrating tablets is the disintegration time. Fast disintegrating tablets were prepared firstly using different excipients (binders and superdisintegrants) and then evaluated for various parameters like friability, hardness, and disintegration time to select the best combination for formulation of fast disintegrating tablets. The combination with the lowest disintegration time, optimum hardness, and friability was selected for further study. 

*Optimization of Superdisintegrant Sodium Starch Glycolate (Primogel, Explotab).* For tablets and capsules which require rapid disintegration, the inclusion of the right superdisintegrant and in its optimum concentration is a prerequisite for optimal bioavailability. Superdisintegrants decrease disintegration time which in turn enhances drug dissolution rate. Thus, the proper choice of superdisintegrant its consistency of performance are of critical importance to the formulation of rapidly disintegrating dosage forms.

Formulation F1–F6 was prepared to study the effect of type and concentration of superdisintegrants in Table 1. Tablets were prepared by direct compression technique. Weighed quantity of Cetirizine Hydrochloride with different concentration of superdisintegrant along with excipients was mixed in geometric progression in a dry and clean mortar. Then the blend was passed through sieve number 60 for direct compression. The powder blend was then compressed into tablets using 8 mm punch in multi punch tablet compression machine (Dhiman Industries, India).

*Optimization of Polyvinylpyrrolidone (PVP K-30) or Microcrystalline Cellulose (Avicel PH-102) as Binder along with Optimized Concentration of Superdisintegrant*. Tablets were prepared by direct compression technique. The composition of fast disintegrating tablet is shown in Table 2. Weighed quantity of Cetirizine Hydrochloride with optimized concentration of Sodium Starch Glycolate along with different concentration of binders (PVP K-30, MCC) along with excipients was mixed in geometric progression in a dry and clean mortar. Then the blend was passed through sieve number 60 for direct compression. The powder blend was then compressed into tablets using 8 mm punch in multi punch tablet compression machine (Dhiman Industries, India).

### 2.3. Final Formulation of Cetirizine Hydrochloride Fast Disintegrating Tablets by Direct Compression Method

Fast disintegrating tablets of Cetirizine Hydrochloride were prepared by direct compression method according to the formula given in Table 3. Weighed quantities of Cetirizine Hydrochloride along with optimized concentration of superdisintegrant and binder along with excipients were mixed in geometric progression in a dry and clean mortar. Then the blend was passed through sieve no. 60 for direct compression. The powder blend was then compressed into tablets using 8 mm punch in multi punch tablet compression machine. These fabricated tablets were evaluated.

### 2.4. Evaluation Parameters

#### 2.4.1. Weight Variation

Twenty tablets were selected, weighed on digital weighting balance (Ohaus, USA) and average weight was determined. Then individual tablets were weighed and the individual weight was compared with an average weight as given in Table 4 [9].

#### 2.4.2. Thickness

Thickness of tablets was determined using Vernier Caliper (Indian caliper industries, Ambala, India). Three tablets from each batch were used and an average value was calculated [9].

#### 2.4.3. Hardness

The crushing strength of the tablets was measured using a Monsanto Hardness Tester (Perfit). Three tablets from each formulation batch were tested randomly and the average reading was noted. The hardness is measured in kg/cm^2^ [11].

#### 2.4.4. Friability

Ten tablets were weighed and placed in a Roche Friabilator (Veego, India) and the equipment was rotated at 25 rpm for 4 min. The tablets were taken out, de-dusted and reweighed. The percentage friability of the tablets was measured as per the following formula [12]

(1) $$\begin{array}{c} \text{Percentage}\;\;\text{friability}=\frac{\text{Initial}\;\text{weight}-\text{Final}\;\text{weight}}{\text{Initial}\;\text{weight}}\times 100. \end{array}$$

#### 2.4.5. In-Vitro Disintegration Test

The test was carried out on 6 tablets using Digital Tablet Disintegration Tester (Veego, India). Distilled water at 37°C ± 2°C was used as a disintegration media and the time in second taken for complete disintegration of the tablet with no palable massremaining in the apparatus was measured in seconds [13]. 

#### 2.4.6. Wetting Time

A petridish containing 6 mL of distilled water was taken. A tablet containing a small quantity of amaranth color was placed on this. Time required for the upper surface of the tablet to become complete red was noted [14].

#### 2.4.7. Drug Content Uniformity

Ten tablets (200 mg) were powdered in mortar pestle and the blend equivalent to 5 mg of Cetirizine Hydrochloride was weighed and dissolved in 100 mL of 6.8 pH phosphate buffer solutions. The solution was sonicated, filtered through whatman filter paper, suitably diluted with 6.8 pH phosphate buffer and the drug content was analyzed by using Double Beam UV Spectrophotometer (UV-1800 Shimadzu) at 230 nm respectively. Each sample was analyzed in triplicate.

#### 2.4.8. In Vitro Dissolution Study

The release of from formulated FDTs was determined using USP eight stage dissolution testing apparatus—2 (paddle method) (Lab, India). The dissolution test was performed using 500 mL of phosphate buffer solution, pH 6.8 at 37 ± 0.5°C and 50 rpm. A sample (5 mL) of the solution was withdrawn from the dissolution apparatus at specific time intervals and the samples were replaced with fresh dissolution medium. The samples were filtered through Whatman filter paper. Absorbance of these solutions was measured at 230 nm using a Double Beam UV Spectrophotometer (UV-1800 Shimadzu). Cumulative percentage (%) of drug release was calculated using standard plot of Cetirizine Hydrochloride [15].

#### 2.4.9. Drug-Excipient Compatibility Studies

These studies were performed in order to confirm the drug-excipient interaction. These studies mainly include FTIR Spectroscopy. FTIR spectra of pure drugs and formulated FDT containing drug were recorded on FTIR Spectrophotometer (Bruker, USA). The scanning range was from 4000 to 600 cm^−1^ and the resolution was 1 cm^−1^. The scans were evaluated for presence of principal peaks of drug, shifting and masking of drug peaks, and appearance of new peaks due to excipient interaction. This spectral analysis was employed to check the compatibility of drugs with the excipients used [16].

#### 2.4.10. Accelerated Stability Studies

The selected formulations were closely packed in aluminum foils and then stored at 40 ± 2°C/75% RH ± 5% in stability chamber for 1 month and evaluated for their physical appearance, drug content, percent friability, and in vitro disintegration time at intervals of the 15th and 30th days [17].

## 3. Results and Discussion

The present investigation was undertaken to formulate and evaluate fast disintegrating tablets of Cetirizine Hydrochloride by direct compression method using Sodium Starch Glycolate as a superdisintegrant and Mannitol as directly compressible diluent and Sodium Saccharin was used to enhance palatability. Avicel PH 102 was included in the formulation as a disintegrant and a binder. This grade of Microcrystalline Cellulose is granular in nature and thus displays excellent flow properties. To impart pleasant taste and improve mouth feel, Sodium Saccharin was included as sweetening agent. Sodium Stearyl Fumarate was employed as a lubricant instead of Magnesium Stearate not only because of the metallic taste of the latter, but also due to its water solubility and directly compressible features.

### 3.1. Optimization of Superdisintegrant Sodium Starch Glycolate (Primogel, Explotab)

Superdisintegrants are generally used by formulation scientists for developing FDTs or for improvement of solubility for drugs. The primary requirement for such dosage forms is quicker disintegration. The amount of superdisintegrants was optimized in the formulation of FDTs. The total 6 formulations (F1–F6) were prepared using different concentration of Sodium Starch Glycolate to study its effect on disintegration time. The results for optimization of superdisintegrant concentration in FDTs by direct compression method are shown in Table 5.

From the evaluation parameters, it was observed that *4% Sodium Starch Glycolate* was the optimum concentration for rapid tablet disintegration on the basis of the least disintegration time observed with F3 formulation. The superdisintegrant action of Sodium Starch Glycolate resulted in hydrophilicity and swelling which in turn causes rapid disintegration. It absorbs water rapidly and swells in water to the extent of 200–300% and disintegrates rapidly. Sodium Starch Glycolate is used as superdisintegrant in tablet formulation at a concentration of 4–6%. Above 8% disintegration times may actually increase due to gelling and its subsequent viscosity producing effects.

### 3.2. Optimization of Polyvinylpyrrolidone (PVP K-30) or Microcrystalline Cellulose (Avicel PH-102) as Binder along with Optimized Concentration of Superdisintegrant

The binders such as Polyvinylpyrrolidone (PVP K-30) or Microcrystalline Cellulose were optimized with superdisintegrant concentration to further study the effect of binders on the disintegration time as well as on hardness and friability of tablets of the formulation. Total 14 formulations (F1–F14) were prepared using different concentration of Polyvinylpyrrolidone (PVP K-30) or Microcrystalline Cellulose to study its effect on disintegration time of formulations. The results for optimization of different binder in FDTs by direct compression method are shown in Table 6.

From the evaluation parameters, it was observed that disintegration time of the formulation was further decreased and tablet hardness, friability with in IP limits. The least disintegration time was observed in F8 formulation, that is, 1% MCC, as compared to F2 formulation, that is, 2% PVP K-30. Water soluble materials such as PVP K-30 tend to dissolve rather than disintegrate, while insoluble materials like MCC generally produce rapidly disintegrating tablets. Due to the presence of porous morphology, liquid is drawn up or “wicked” into these pathways through capillary action and ruptures the interparticulate bonds causing the tablet to break apart. Therefore *1% Microcrystalline Cellulose* was selected as optimum binder concentration selected for final formulation of Cetirizine Hydrochloride FDT.

### 3.3. Evaluation Parameters for Cetirizine Hydrochloride Fast Disintegrating Tablet

Final formulation of Cetirizine Hydrochloride FDT was tested for all the official tests of tablet and was found to be within limits as shown in Table 7. Percent weight variation was well within the acceptable limit for uncoated tablets as per Indian Pharmacopoeia. It is well known to formulation scientists that the tablets with more hardness show longer disintegration time. Since mechanical integrity is of paramount importance in successful formulation of FDTs, hence the hardness of tablets was determined. The friability of Cetirizine Hydrochloride FDT was less than 1% which is acceptable according IP criteria. The content uniformity of the prepared Cetirizine Hydrochloride FDT was complied with IP specifications. No tablet from ten tablets lies out of the range of 85–115% of the label claim. These results indicated that the dosage form had uniform distribution and proper dose of the active ingredient. The wetting time and disintegration time were practically good for formulation. According to IP, the dispersible tablet must disintegrate within 3 minutes, but the formulated FDTs showed low DT indicating suitability of formulation for mouth dissolving tablet.

### 3.4. In Vitro Dissolution Study

**Figure 1:**
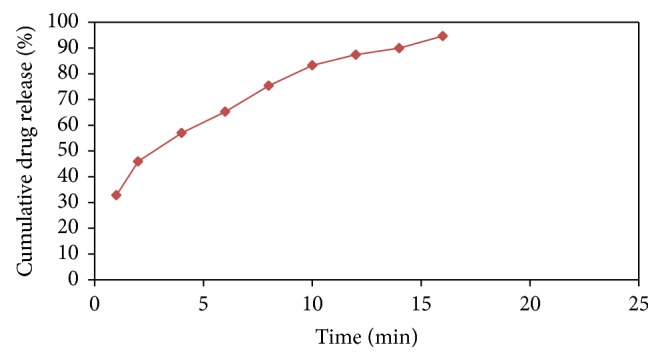
In-vitro Dissolution Profile of Cetirizine Hydrochloride FDT.

In vitro dissolution studies showed that more than 50% of the drug was released from the formulation within 5 minutes. The rapid drug dissolution might be due to easy breakdown of particle by superdisintegrant action. From in vitro dissolution data, it was observed that 94.74 ± 2.48% of Cetirizine Hydrochloride released in 16 minutes as shown in Figure 1 indicates that the tablet complies as per IP specifications, that is, 85%–110%.

### 3.5. Drug-Excipient Compatibility Studies

**Figure 2:**
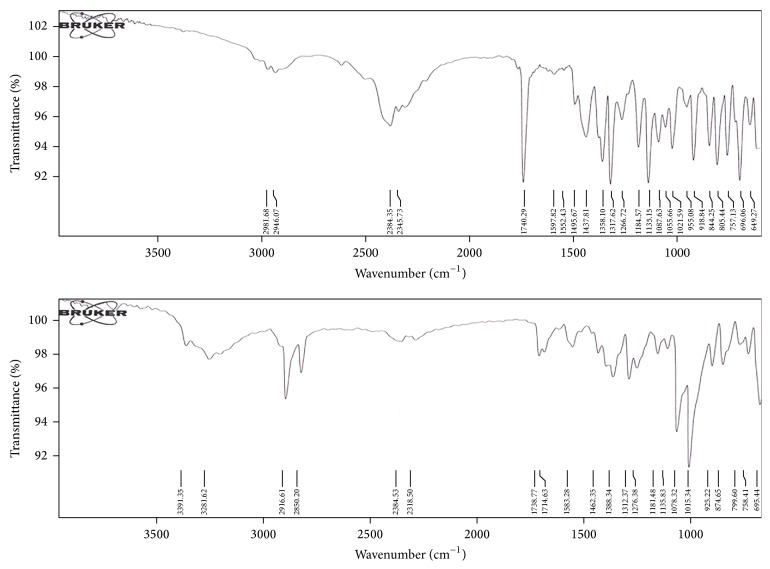
FTIR Spectra of Cetirizine Hydrochloride (Pure Drug) V/S FTIR Spectra of Cetirizine Hydrochloride FDT.

The results obtained with IR studies showed that there was no interaction between the drug and other excipients used in the formulation. The FTIR of Cetirizine Hydrochloride had shown intense band at 757.13 cm^−1^, 1317.62 cm^−1^, 1055.66 cm^−1^ and 1184.57 cm^−1^ corresponding to the presence of functional groups such as aliphatic chlorocompound, carboxylic acid, alkyl substituted ether and tertiary amine. The FTIR of Cetirizine Hydrochloride FDT formulation shown intense bands at 758.41 cm^−1^, 1312.37 cm^−1^, 1078.32 cm^−1^ and 1181.48 cm^−1^ indicates no change in the functional groups such as aliphatic chlorocompound, carboxylic acid, alkyl substituted ether, and tertiary amine confirming undisturbed structure of Cetirizine Hydrochloride, which indicates no drug-excipient interaction as shown in Figure 2.

### 3.6. Accelerated Stability Studies

In the present study, stability studies were carried out on formulated FDTs (formulated in three primary batches), wrapped in aluminium foil to prevent the formulation from exposure to light to simulate the aluminum packaging, that is, Alu Alu packing, of drug products, and stored in air-tight containers which is impermeable to solid, liquid, and gases, under the following condition for onemonth period as prescribed by ICH guidelines for accelerated stability study. During the stability studies, the product is exposed to normal condition of temperature and humidity. However the studies will take a longer time and hence it would be convenient to carry out accelerated stability studies, where the product is stored under extreme condition of temperature and humidity. The stability data of formulation are shown in Tables 8 and 9 as given.

Result is obtained after 1 month of stability studies at room temperature and at ambient humidity.

The result of the stability study indicated that there were not many differences observed in hardness, disintegration time, drug content uniformity, and friability before and after the storage period at room temperature and at ambient humidity, but, at temperature of 40 ± 2°C/75% RH ± 5% relative humidity, hardness was increased with time, prolonging the DT of the tablet; the probable reason was loss of moisture from tablets, but, in all cases, DT is within the specified IP limit (within 3 min). This indicates that formulation is fairly stable at both storage conditions.

## 4. Conclusion

Fast disintegrating tablet is a promising approach with a view of obtaining faster action of the drug and would be advantageous in comparison to currently available conventional dosage forms. The FDT dosage form had a good balance over disintegration time and mechanical strength. The prime objective of the study was to develop Cetirizine Hydrochloride fast disintegrating tablet by using commonly available excipients and conventional technology. From the above study, it was concluded that, by employing commonly available pharmaceutical excipients, such as superdisintegrants, hydrophilic and swellable excipients and proper filler, a fast disintegrating tablet of Cetirizine Hydrochloride can be developed which can be commercialized.

## Figures and Tables

**Table 1:** Formula for 1 tablet (200 mg) of different concentration of Sodium Starch Glycolate (data in mg).

Sr. number	Ingredients	F1	F2	F3	F4	F5	F6
1	Cetirizine Hydrochloride	5	5	5	5	5	5
2	Sodium Starch Glycolate	2 (1%)	4 (2%)	8 (4%)	12 (6%)	16 (8%)	20 (10%)
3	Polyvinylpyrrolidone K-30	4	4	4	4	4	4
4	Magnesium Stearate	3	3	3	3	3	3
5	Talc	3	3	3	3	3	3
6	Sodium Saccharin	5	5	5	5	5	5
7	Mannitol	178	176	172	168	164	160

**Table 2:** Formula for 1 tablet (200 mg) for the optimization of Polyvinylpyrrolidone K-30 or Microcrystalline Cellulose with optimized concentration of Sodium Starch Glycolate.

Formula number	Contents							
	Cetirizine Hydrochloride (mg)	SSG (mg)	PVP K-30 (mg)	MCC (mg)	Sodium Stearyl Fumarate (mg)	Talc (mg)	Sodium Saccharin (mg)	Mannitol (mg)
F1	5	8	2	—	2	2	5	176
F2	5	8	4	—	2	2	5	174
F3	5	8	6	—	2	2	5	172
F4	5	8	8	—	2	2	5	170
F5	5	8	10	—	2	2	5	168
F6	5	8	12	—	2	2	5	166
F7	5	8	14		2	2	5	164
F8	5	8	—	2	2	2	5	176
F9	5	8	—	4	2	2	5	174
F10	5	8	—	6	2	2	5	172
F11	5	8	—	8	2	2	5	170
F12	5	8	—	10	2	2	5	168
F13	5	8	—	12	2	2	5	166
F14	5	8	—	14	2	2	5	164

**Table 3:** Formula of Cetirizine Hydrochloride FDT prepared by direct compression method (data in mg).

Sr. number	Ingredients	Formula for 1 tablet (200 mg)	Formula for 110 tablets (200 mg)
1	Cetirizine Hydrochloride	5	550
2	Sodium Starch Glycolate	8	880
3	Microcrystalline Cellulose	2	220
4	Sodium Stearyl Fumarate	4	440
5	Talc	2	220
6	Sodium Saccharin	8	880
7	Mint flavor	8	880
8	Mannitol	163	17930

**Table 4:** Weight variation specification as per Indian Pharmacopoeia (IP).

Average weight of tablet	% deviation
80 mg or less	±10
More than 80 mg but less than 250 mg	±7.5
250 mg or more	±5

**Table 5:** Evaluation parameters for the optimization of Sodium Starch Glycolate.

	Evaluation parameters	F1 (1% SSG)	F2 (2% SSG)	F3 (4% SSG)	F4 (6% SSG)	F5 (8% SSG)	F6 (10% SSG)
1	Weight variation (IP)	Passed	Passed	Passed	Passed	Passed	Passed
2	Friability (%)	0.8	0.8	0.1	0.3	0.1	0.1
3	∗Hardness (Kg/cm^2^) ± SD	2.2 ± 0.57	1.6 ± 0.28	1.5 ± 0.28	1.5 ± 0.32	2.0 ± 0.57	1.8 ± 0.28
4	∗∗Disintegration time (Sec) ± SD	80 ± 2.34	59 ± 6.67	40 ± 2.63	48 ± 6.38	138 ± 7.39	95 ± 6.97

^*^Average of three determinations, ^**^average of six determinations.

Bold font refers to disintegration time of 4% SSG.

**Table 6:** Evaluation parameters for the optimization of Polyvinylpyrrolidone (PVP K-30) or Microcrystalline Cellulose as binder with optimized concentration of Sodium Starch Glycolate.

Formula number	Evaluation parameters			
	Weight variation (IP)	Friability (%)	∗Hardness (Kg/cm^2^) ± SD	∗∗Disintegration time (Sec) ± SD
F1	Passed	0.1	2.2 ± 0.28	60 ± 1.78
F2	Passed	0.2	1.8 ± 0.28	49 ± 1.67
F3	Passed	0.5	2.0 ± 0.00	69 ± 2.89
F4	Passed	0.3	3.2 ± 0.76	83 ± 2.40
F5	Passed	0.3	1.6 ± 0.50	90 ± 5.16
F6	Passed	0.8	2.5 ± 0.50	120 ± 5.77
F7	Passed	0.8	2.0 ± 0.00	145 ± 5.43
F8	Passed	0.1	1.5 ± 0.50	37 ± 3.13
F9	Passed	0.1	1.5 ± 0.28	47 ± 1.34
F10	Passed	0.2	1.5 ± 0.28	62 ± 1.10
F11	Passed	0.1	1.8 ± 0.28	75 ± 1.32
F12	Passed	0.1	1.5 ± 0.28	82 ± 2.08
F13	Passed	0.1	1.8 ± 0.28	96 ± 1.84
F14	Passed	0.1	1.8 ± 0.28	105 ± 2.73

^*^Average of three determinations, ^**^average of six determinations.

Bold font refers to disintegration time of 2% PVP K-30 and disintegration time of 1% MCC.

**Table 7:** Evaluation parameters for Cetirizine Hydrochloride FDT.

Sr. number	Evaluation parameters	Results
1	Weight variation (IP)	Passed
2	∗Thickness (mm) ± SD	3.65 ± 0.09
3	∗Hardness (Kg/cm^2^) ± SD	1.5 ± 0.58
4	Friability (%)	0.5
5	∗∗Disintegration time (sec) ± SD	35 ± 4.02
6	∗Wetting time (sec) ± SD	23 ± 1.15
7	∗Drug content uniformity ± SD	93.33 ± 1.53

^*^Average of three determinations, ^**^average of six determinations.

**Table 8:** Accelerated stability studies of Cetirizine Hydrochloride FDT stored at 40 ± 2°C/75% RH ± 5%.

	Three primary batches								
Time interval	Day 0			The 15th day			The 30th day		
Evaluation parameters	B-1	B-2	B-3	B-1	B-2	B-3	B-1	B-2	B-3
∗Hardness (Kg/cm^2^) ± SD	1.3 ± 0.58	1.2 ± 0.29	1.8 ± 0.29	1.9 ± 0.29	2.0 ± 0.29	1.5 ± 0.00	3.0 ± 0.5	2.5 ± 0.29	1.7 ± 0.29
Friability (%)	0.1	0.4	0.6	0.3	0.4	0.2	0.1	0.2	0.5
Drug content ± SD	100.8 ± 3.36	95.6 ± 2.34	93.8 ± 1.24	98.5 ± 2.14	99.4 ± 2.67	90.42 ± 3.64	92.8 ± 1.98	99 ± 1.65	97.6 ± 3.63
∗∗Disintegration time (sec) ± SD	37 ± 4.79	40 ± 3.64	35 ± 2.27	44 ± 2.06	46 ± 2.18	39 ± 3.09	45 ± 3.43	50 ± 3.27	44 ± 2.23

^*^Average of three determinations/batch. ^**^average of six determinations/batch.

**Table 9:** Accelerated stability studies of Cetirizine Hydrochloride FDT at room temperature at ambient humidity.

	Three primary batches								
Time interval	Day 0			The 15th day			The 30th day		
Evaluation parameters	B-1	B-2	B-3	B-1	B-2	B-3	B-1	B-2	B-3
∗Hardness (Kg/cm^2^) ± SD	1.3 ± 0.58	1.2 ± 0.29	1.8 ± 0.29	1.4 ± 0.50	1.5 ± 0.00	1.7 ± 0.29	1.3 ± 0.5	1.5 ± 0.29	1.5 ± 0.29
Friability (%)	0.1	0.4	0.6	0.3	0.2	0.2	0.4	0.2	0.3
∗Drug content ± SD	100.8 ± 3.36	95.6 ± 2.34	93.8 ± 1.24	99.5 ± 2.14	94.5 ± 2.67	94.8 ± 1.23	98.3 ± 1.98	95.4 ± 1.65	95.7 ± 3.63
∗∗Disintegration time (sec) ± SD	37 ± 4.79	40 ± 3.64	35 ± 2.27	35 ± 3.60	42 ± 4.44	38 ± 2.18	40 ± 3.64	38 ± 1.05	41 ± 1.31

^*^Average of three determinations/batch, ^**^average of six determinations/batch.
